# The seed of *Litchi chinensis* fraction ameliorates hippocampal neuronal injury in an Aβ_25-35_-induced Alzheimer’s disease rat model via the AKT/GSK-3β pathway

**DOI:** 10.1080/13880209.2019.1697298

**Published:** 2019-12-27

**Authors:** Yueshan Sun, Anguo Wu, Xiu Li, Dalian Qin, Bingjin Jin, Jian Liu, Yong Tang, Jianming Wu, Chonglin Yu

**Affiliations:** a Department of Pharmacology, School of Pharmacy, Southwest Medical University, Luzhou, China; b Institute of Cardiovascular Research, The Key Laboratory of Medical Electrophysiology, Southwest Medical University, Luzhou, China; c Department of Anatomy and Histology and Embryology, Chengdu Medical College, Chengdu, China; d Department of Human Anatomy, Chengdu Medical Collage, Chengdu, China

**Keywords:** *Litchi chinensis* seed fraction, neuroprotection, AD, botanical drug, cognitive function, beta-amyloid, tau, AKT, GSK-3β

## Abstract

**Context:**

The seed of *Litchi chinensis* Sonn., a famous traditional Chinese medicine, was recently reported to enhance cognitive function by inhibiting neuronal apoptosis in rats.

**Objective:**

We determined whether the seed of *Litchi chinensis* fraction (SLF) can ameliorate hippocampal neuronal injury via the AKT/GSK-3β pathway.

**Materials and methods:**

We established Alzheimer’s disease (AD) model by infusing Aβ_25-35_ into the lateral ventricle of Sprague–Dawley (SD) rats and randomly divided into five groups (*n* = 10): sham, donepezil and SLF (120, 240 and 480 mg/kg/d). Rats were treated by intragastric administration for 28 consecutive days. Spatial learning and memory were evaluated with Morris water maze, while protein expression of AKT, GSK-3β and tau in the hippocampal neurons was measured by Western blotting and immunohistochemistry.

**Results:**

On the fifth day, escape latency of the AD model group was 45.78 ± 2.52 s and that of the sham operative group was 15.98 ± 2.32 s. SLF could improve cognitive functions by increasing the number of rats that crossed the platform (*p* < 0.01), and their platform quadrant dwell time (*p* < 0.05). The protein expression level of AKT was upregulated (*p* < 0.001), while that of GSK-3β and tau (*p* < 0.01) was remarkably downregulated in the hippocampal CA1 area.

**Discussion and conclusions:**

To our knowledge, the present study is the first to show that SLF may exert neuroprotective effect in AD rats via the AKT/GSK-3β signalling pathway, thereby serving as evidence for the potential utility of SLF as an effective drug against AD.

## Introduction

Alzheimer’s disease (AD) is a typical progressive neurodegenerative disorder characterized by a deterioration in memory, learning and other higher cognitive functions (Chen et al. 2014). There is increasing evidence that abnormal sediments of amyloid β (Aβ) peptide and intracellular tau-containing neurofibrillary tangles (NFTs) are two primary pathological hallmarks of AD (Carr 2015; Akhtar et al. 2016; Li et al. 2016). Aβ is an insulin-suppressing agent that inhibits insulin signalling by inhibiting the interconnection between insulin and its idiosyncratic acceptor, ultimately causing insulin resistance (Zhao et al. 2008; Carr 2015). The weakened insulin signalling pathways antagonize the insulin receptors, resulting in dysfunctions in the downstream pathways (PI3K/AKT, MAPK and Wnt) (Lee et al. 2011; Shen et al. 2011; Pramojanee et al. 2014) and a series of characteristic lesion changes in AD, including tau protein hyperphosphorylation, Aβ accumulation and neuronal apoptosis (Heras-Sandoval et al. 2012; Yang et al. 2012). Therefore, Aβ accumulation plays a key role in the pathological process involved in the PI3K/AKT/GSK-3β pathway.

The PI3K/AKT/GSK-3β pathway plays a key role in the maintenance of neuronal network, cell survival and longevity. Disturbance of this signalling pathway is the pivotal mechanism underlying the pathology of neurodegenerative diseases, such as AD (Kitagishi et al. 2014; Zhang et al. 2014; Qi et al. 2015). Moreover, neuronal apoptosis may be affected by the insulin signalling pathway through the inhibition of the pro-apoptosis protein families, including Bcl-2-associated death (BAD) promoter, and its involvement in AKT and GSK-3β (Li et al. 2009; Gomez-Sintes et al. 2011). Once the anti-apoptotic Bcl-2 family proteins are inhibited by BAD, neuronal apoptosis will occur (Cerioni and Cantoni 2010). Insulin can also inhibit BAD-induced neuronal apoptosis by triggering PI3K/AKT and inactivating GSK-3β (Kamada et al. 2007). In contrast, Aβ accumulation in AD may inhibit PI3K/AKT to accelerate the expression of pro-apoptosis genes, such as GSK-3β, BAD and NF-κB and result in neuronal apoptosis (Ma et al. 2009). Recent studies on the pathogenesis of AD indicate that neuronal apoptosis plays a key role in neuronal injury or loss (Crews and Masliah 2010). These revelations suggest that some materials may exhibit neuron protection properties via insulin signals and the PI3K/AKT/GSK-3β pathway.

Recently, many compounds derived from traditional Chinese medicines (TCMs), including gypenoside (Meng et al. 2016), senegenin (Jesky and Chen 2015), ginsenoside Rb1 (Dong et al. 2017), arjunolic acid (Yaidikar and Thakur 2015), ginsenoside Rd (Liu et al. 2015a), ginsenoside Rg1 (Xie et al. 2015), tenuifolin and others (Liu et al. 2015b; Sun et al. 2015), have been suggested to display neuroprotective effects. The polyphenol-enriched lychee seed, a prevailing TCM listed in the Compendium of Material Medica, has a beneficial effect on relieving polydipsia (i.e., excessive thirst and one of the initial symptoms of diabetes mellitus) and treating diabetes mellitus for a long period of time. Pharmacological studies have revealed that the seed of *Litchi chinensis* possesses multiple pharmacological activities, such as modulating blood glucose, moderating insulin resistance and sensitivity of epigallocatechin gallate (Xu et al. 2010; Zhang et al. 2018), and blood lipids (Jaiswal and Kumar 2015), and preventing liver injury (Zhang et al. 2018), oxidation (Jaiswal and Kumar 2015), virus (Guo et al. 2017) and tumour (Zhao et al. 2007) activities. The seeds of *Litchi chinensis* contain remarkable amounts of phenolic compounds such as protocatechuic acid, protocatechuic aldehyde, procyanidin D, (–)-epicatechin, cinnamtannin B1, procyanidin A1, rutin and phlorizin (Man et al. 2016, 2017). In addition, it also contains glycosides such as litchioside D, taxifolin 4′-*O*-β-d-glucopyranoside and kaempferol 7-*O*-neohesperidoside (Xu et al. 2011; Wang et al. 2017). Other categories such as organic acids, flavonoids, amino acids, fatty acids and sugar, are also fundamental chemical components of *Litchi chinensis* seeds (Guo and Pan 2006; Zhang and Zhang 2015). The aqueous extracts of the seed can improve learning and memory in mice (Ye et al. 2013). Recently, we reported that *Litchi chinensis* seed fraction (SLF) improved cognitive function by inhibiting neuronal apoptosis in rats (Wang et al. 2017). However, it remains unclear whether SLF can prevent hippocampal neuronal injury via the AKT/GSK-3β signalling pathway. In the present study, we investigated the effect of SLF on cognitive function *in vivo* and the protein expression of AKT, GSK-3β and tau in hippocampal neurons by immunohistochemistry and Western blotting (WB) analysis.

## Materials and methods

### Preparation of plant extract

*Litchi chinensis* seeds were purchased from the herbal medicine market in Luzhou on 17 September 2017 (Sichuan, China) and were authenticated by Can Tang, a professor at Southwest Medical University (Luzhou, China). A voucher specimen (SWMU-YL-LZH2017091702) was deposited at the Herbarium of Traditional Chinese Medicine, School of Pharmacy, Southwest Medical University. The dehydrated seeds (1000 g) were crushed and infiltrated with 1000 mL of 70% ethanol for 12 h, and then refined by percolation with 8000 mL 70% ethanol at a percolate speed of 5 mL/min/kg. The total solvent volume of 8370 mL was then evaporated under vacuum and filled to 5000 mL with distilled water. SLF in the percolation was absorbed using D101 macroporous resins at a speed of 6 mL/min, collected by elution with 70% ethanol (800 mL), and enriched to dryness by a rotary vacuum evaporator; 31.75 g dried extract was obtained.

### Experimental animals

This study was carried out according to the recommendations of the Animal Experimentation Ethics Committee of Southwest Medical University and abided by the Guide for the Care and Use of Laboratory Animals published by the US National Institute of Health (NIH Publication, 8th Edition, 2011). Six-month-old specific pathogen free-grade (SPF) male Sprague-Dawley (SD) rats (body weight, 200–240 g) were supplied by the Chengdu Dashuo Experimental Animal Co. Ltd. (certificate no. SCXK201302, Chengdu, Sichuan, China). Rats were housed with free access to commercial water and diet in normal plastic cages at an invariable room temperature (25 °C) under a 12 h light/dark cycle.

### Reagents and antibodies

SLF was prepared at a concentration of 100 mg/mL at the School of Pharmacy, Southwest Medical University (Luzhou, Sichuan, China) using a 1.0 g suspension equivalent to 31.5 g crude material. After purchasing donepezil hydrochloride (lot: 110823A) from Eisai Inc. (Suzhou, Jiangsu, China), a 0.1 mg/mL concentration was prepared. Aβ_25-35_ (lot: Y-0044) was acquired from BIOSS Biotech Co., Ltd. (Beijing, China). Anti-AKT and anti-GSK-3β antibodies were purchased from Abcam (Cambridge, MA, USA). Anti-tau antibody was obtained from American Bioworld Co., Ltd. (Dublin, OH, USA). Anti-GAPDH antibody was purchased from Kangchen Bio-tech Inc. (Shanghai, China). The brain stereotaxic apparatus was purchased from Huaibei Zhenhua Biological Equipment Co., Ltd. (Anhui, Huaibei, China). The Morris water maze was purchased from Chengdu Taimeng Technology Co., Ltd. (Chengdu, Sichuan, China). An S70 Inverted microscope was purchased from Leica DMIRB (Rueil-Malmaison, France). The ChemiDocXRS gel imaging and analysis system was purchased from Bio-Rad Laboratories, Inc. (Berkeley, CA, USA). SP-9000 immunohistochemistry kit was purchased from Beijing ZSGB Biological Engineering Co., Ltd. (Beijing, China). ECL Western detection reagents were from 4A Biotech Co., Ltd. (Beijing, China).

### Morris water maze test

The Morris water maze is the most frequently used device in behavioural neuroscience. The standard detection procedure has been described previously (Deng et al. 2016; Tabrizian et al. 2018). Briefly, the experiment was carried out in a round white pool (size, 94 cm in diameter and 31 cm deep), filled with water to a depth of 30 cm at a temperature of ∼25 °C. A 25-cm^2^ Plexiglas square was used as the escape platform and was placed in the centre of one quadrant in the pool, ∼15 cm from the pool’s edge, and submerged 1 cm beneath the water surface.

*Hidden-platform test*: The pool was divided into four equal quadrants. Experimental rats were trained on a hidden platform with four trials (120 s per trial plus a 30-min interval between trials) daily for five consecutive days. The time taken by the rat to find the platform was recorded by an online image video tracking system and was within 120 s, similar to the escape latency.

*Spatial probe test*: The platform was first removed from the pool after completion of the hidden platform test. Rats were then placed on the quadrant farthest from the primary platform, and the number of rats crossing the platform and the time spent in the target quadrant were recorded by a video tracking system.

### Establishment of the AD model

In the current study, the AD animal model was established per our previously described method (Ma et al. 2013; Wang et al. 2017). In brief, normal male SD rats were first stereotaxically injected with 10 μg aggregated Aβ_25-35_ in the lateral ventricle at accurate positions based on the dorsoventral (DV), mediolateral (ML) and anteroposterior (AP) coordinates (respectively –2.8 mm, –2.2 mm and –3.0 mm), to locate the CA1 subregion of their hippocampus (Facchinetti et al. 2018). Rats were then evaluated using the Morris water maze test according to the following criterion (Ma et al. 2013). Rats that could cross the platform between 40 s and 120 s were selected for a further study. Treatment was initiated on day 21 following Aβ_25-35_ injection.

### Drug preparation and administration

SLF was first ground with 1% Tween-80 (mass ratio with SLF). Thereafter, water was added to prepare a concentration of 100 mg/mL. Sham rats and AD rats were selected for the Morris water maze studies three weeks after the operation according to a standard reference (Ma et al. 2013). Rats with AD were randomly divided into five groups (*n* = 10 rats per group) and treated with NS (negative control), donepezil 0.42 mg/kg (positive control) and SLF 120, 240 and 480 mg/kg by intragastric administration once per day for 28 consecutive days. Rats in the sham group were given an equal volume of NS as the negative control.

### Immunohistochemical staining

Rats were anaesthetized by an intraperitoneal injection of 1.0% pentobarbital and their brains were perfused with 4% paraformaldehyde for fixing 24 h after NS or drug treatments. After that, brains were removed, and the brain tissues were weighed, fixed in 10% neutral-buffered formalin, dehydrated and embedded in paraffin to create coronal microtome sections (4–5 μm). The sections were then stained immunohistochemically according to previous methods (Focke et al. 2018). A subsequent pathological study (AKT, GSK-3β and tau) was performed via observation with a light microscope (×400). The integrated optical density (IOD) of AKT, GSK-3β and tau were analysed by Image Pro Plus 6.0 software.

### Western blotting analysis

Western blotting for the detection of the protein expression in the CA1 region of hippocampal slice was performed as described previously (Seibenhener and Wooten 2012). Briefly, the CA1 region of the hippocampal slice was isolated under a stereomicroscope (McNair et al. 2010; Yuanxiang et al. 2014). The isolated CA1 regions were homogenized with cold phosphate-buffered saline (PBS) followed by homogenization with a tissue homogenizer (Sceintz-48, Ningbo, China) to create small pieces. Proteins were then extracted with 1× RIPA buffer (0.5% NP-40, 50 mM Tris–HCl, 120 mM NaCl, 1 mM EDTA, 0.1 mM Na_3_VO_4_, 1 mM NaF, 1 mM PMSF and 1 μg/mL leupeptin, pH 7.5) containing proteasome inhibitor. After centrifugation at 12,000×*g* for 15 min at 4 °C, protein concentrations were measured by the BCA method and an equal amount of protein (30 μg) was electrophoresed on 7.5% SDS-acrylamide gels. Following electrophoresis, the proteins were transferred to a nitrocellulose membrane using an electric transfer system at 250 mA for 90 min. Non-specific binding was blocked with 5% skim milk in TBST buffer (5 mM Tris–HCl, 136 mM NaCl and 0.1% Tween-20, pH 7.6) for 1 h. The blots were incubated with antibodies against GSK-3β (1:800), AKT (1:300) or GAPDH (1:10,000) overnight at 4 °C, washed three times with 1× PBST, incubated for 1 h at room temperature with a 1:3000 dilution of horseradish peroxidase-labelled anti-rabbit or anti-mouse IgG, and washed three times with 1× PBST. The membranes were developed by incubation with ECL Western detection reagents. The protein expression levels of GSK-3β and AKT were detected using the Chemi-Doc image analyser (Bio-Rad, Hercules, CA, USA) and the relative rates of GSK-3β/GAPDH and AKT/GAPDH were calculated.

### Statistical analysis

All data are expressed as mean ± standard deviation (SD). Statistical differences among the data were analysed by one-way univariate analysis of variance (ANOVA) for comparison of multiple groups. *p < 0.05; **p < 0.01, and ***p < 0.001 were considered to be significant.

## Results

### Successful establishment of the AD model in rats

To study the cognitive function of rats with AD, escape latency was derived for normal rats (sham group, A) and rats with AD (model group, B). On the fifth day of positioning navigation training, escape latency was 15.98 ± 2.32 s for the sham operative group and 45.78 ± 2.52 s for the model group. By using the formula, (B – A)/B%, a value of 65.09% was obtained, ultimately suggesting the successful establishment of the AD model induced by Aβ_25-35_, according to the AD model criteria of (B – A)/B% > 20%.

### SLF improved spatial learning and memory in rats with AD

**Figure 1.**
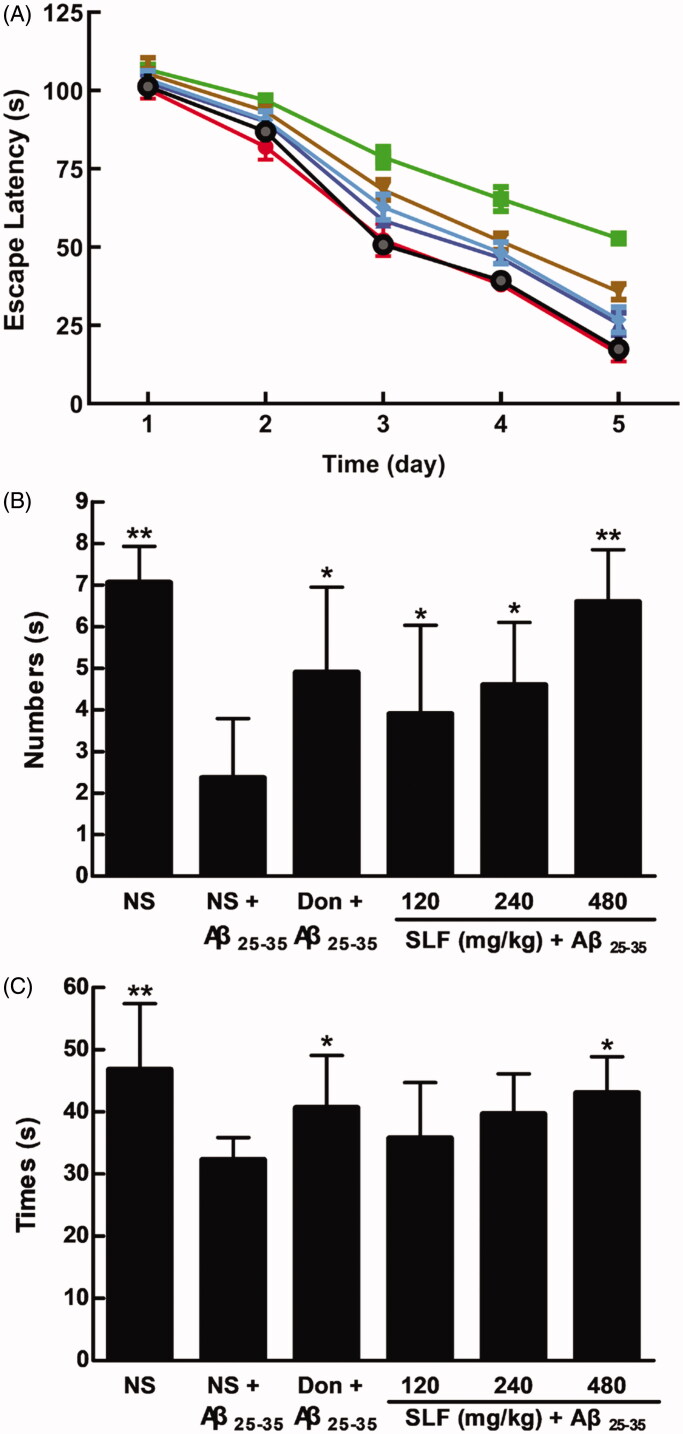
Effects of SLF and donepezil (Don) on spatial learning and memory in sham rats and those with AD induced by Aβ_25-35_. Sham rats were treated with normal saline (NS), and AD rats were treated with NS, Don 0.42 mg/kg, or SLF 120, 240 and 480 mg/kg × 28 days by intragastric administration. The behavioural test was performed using the Morris water maze (A). Escape latency: (●) sham rats treated with normal saline; (■) AD rats treated with normal saline; (▲) donepezil 0.42 mg/kg × 28 days; (▼) SLF 120 mg/kg × 28 days; (♦) SLF 240 mg/kg × 28 days; (^) SLF 480 mg/kg × 28 days. (B) The number of rats that crossed the platform; and (C) platform quadrant dwell time. There were 10 rats in each experimental group. Results are expressed as mean ± SD. *p < 0.05, **p < 0.01 vs. the AD rats treated with NS.

**Figure 2.**
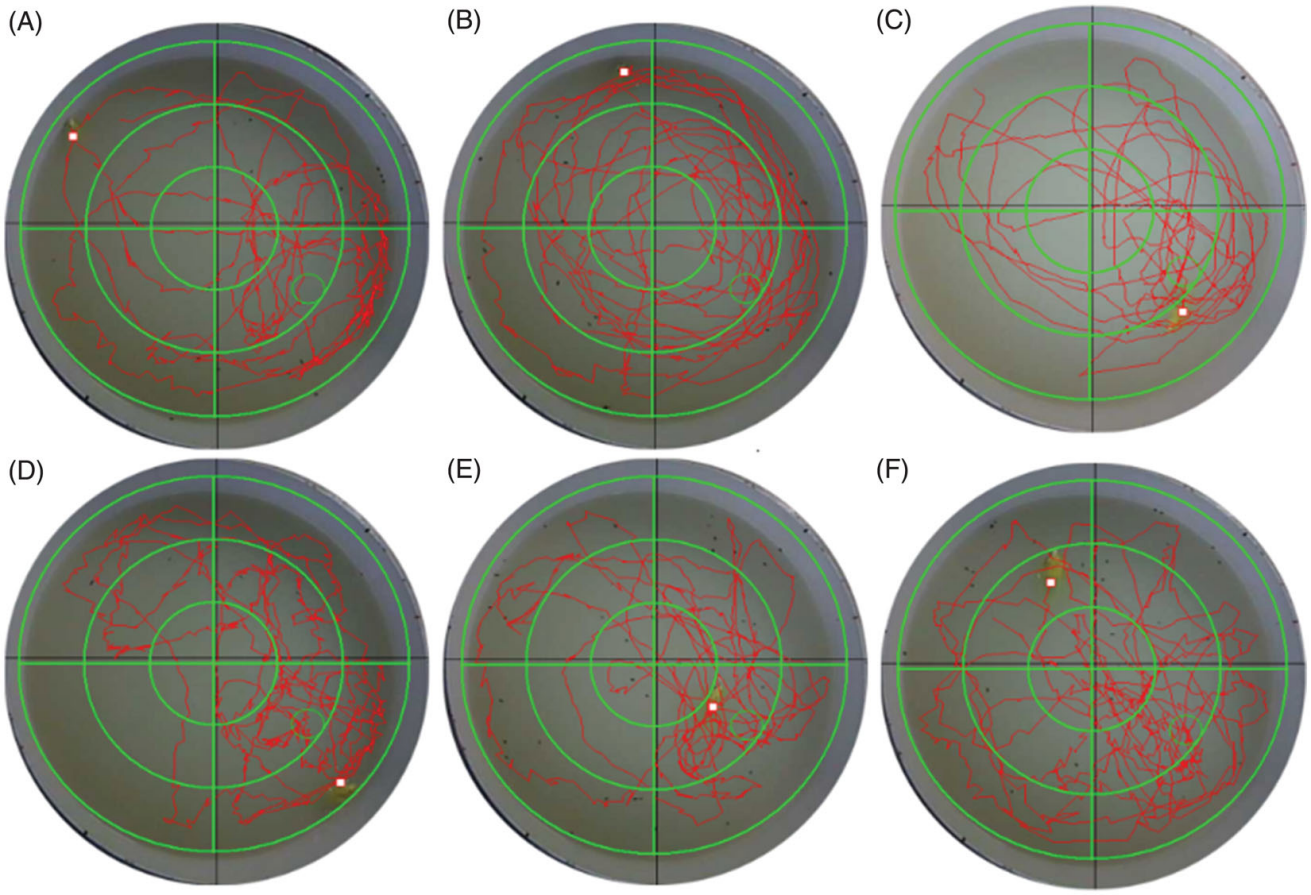
Effects of SLF on the spatial probe ability in sham rats and those with Aβ_25-35_-induced AD. (A) Sham rats were treated with normal saline (NS) and (B–F) AD rats were treated with NS, donepezil 0.42 mg/kg or SLF 120, 240 and 480 mg/kg × 28 days by intragastric administration. The behavioural test was performed using the Morris water maze.

Increasing evidence shows that insulin resistance is associated with the pathology of AD, and prior research has demonstrated that it can be improved with SLF. Therefore, we hypothesized that SLF may be involved in the effect on AD. For this purpose, we examined spatial learning and memory in rats with AD. As shown in Figure 1(A), escape latency of rats with AD was markedly increased relative to that of rats in the sham group. However, administering SLF at doses of 120, 240 and 480 mg/kg, and donepezil at 0.42 mg/kg for 28 days, significantly shortened the escape latency. Similarly, the number of rats crossing the platform (Figure 1(B)) and time spent in the target quadrant (Figure 1(C)) were significantly shortened for rats with AD compared to rats in the sham group (*p* < 0.01). Nonetheless, by administering SLF (120, 240 and 480 mg/kg × 28 days) and donepezil, a significant increase in the numbers of rats crossing the platform (*p* < 0.05 or *p* < 0.01) and the time spent in the target quadrant (*p* < 0.05) was found. Meanwhile, the SLF groups and the donepezil group had reduced percentage of total distance run on the platform quadrant (Figure 2).

### SLF promoted the protein expression of AKT in AD rats by immunohistochemistry

**Figure 3.**
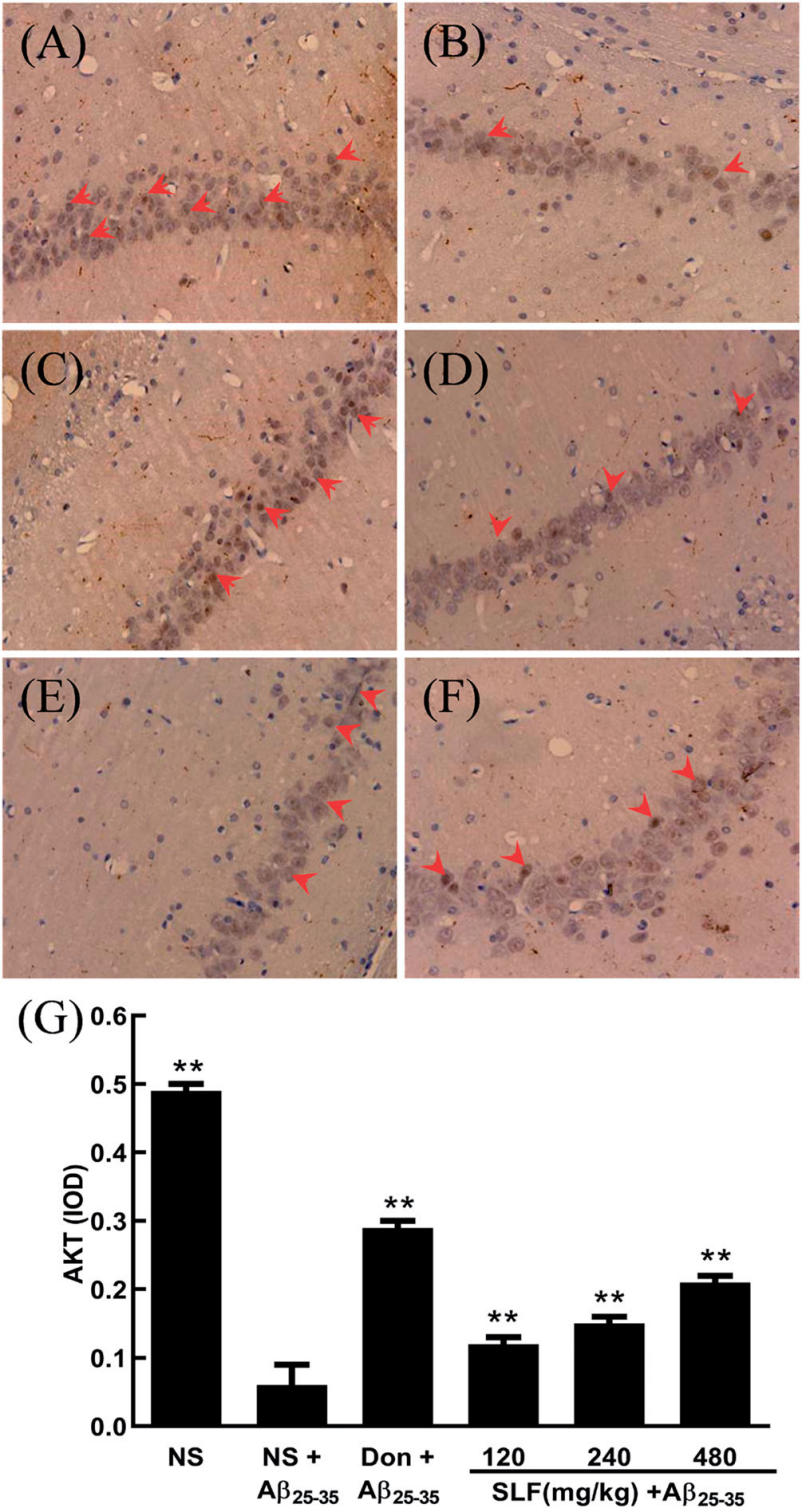
SLF upregulated AKT protein expression in the hippocampal CA1 area of rats with Aβ_25-35_-induced AD by immunohistochemistry (×400). (A) Sham rats treated with NS; (B–F) AD rats treated with NS, donepezil 0.42 mg/kg, SLF 120, 240 and 480 mg/kg by intragastric administration. Ten rats were allocated to each group. The effects of SLF on the integrated optical density of AKT expression in AD rats (G). The results are expressed as mean ± SD (*n* = 10). **p < 0.01, compared to the NS plus Aβ_25-35_ group (normal saline treatment).

AKT kinase plays an important role in neuron proliferation and survival (Hanada et al. 2004). Here, we used immunohistochemistry and the anti-AKT antibody to examine the protein expression of AKT in the hippocampal CA1 area of experimental rats. As shown in Figure 3(A), higher levels of the positive feature of AKT expression were clearly observed in the hippocampal CA1 area of sham rats, with many brown particles scattered in the cytoplasm of the hippocampal tissues (Figure 3(A)) and few brown particles sparsely detected in rats with AD (Figure 3(B)). Moreover, IOD of AKT expression was evidently decreased compared to that of sham rats (*p * < 0.01; Figure 3(G)). However, the number of brown particles was significantly increased after donepezil (Figure 3(C)) or SLF (Figure 3(D–F)) treatment in a dose-dependent manner, aligning with the results for the IOD of AKT expression in Figure 3(G).

### SLF decreased the protein expression of GSK-3β in AD rats by immunohistochemistry

**Figure 4.**
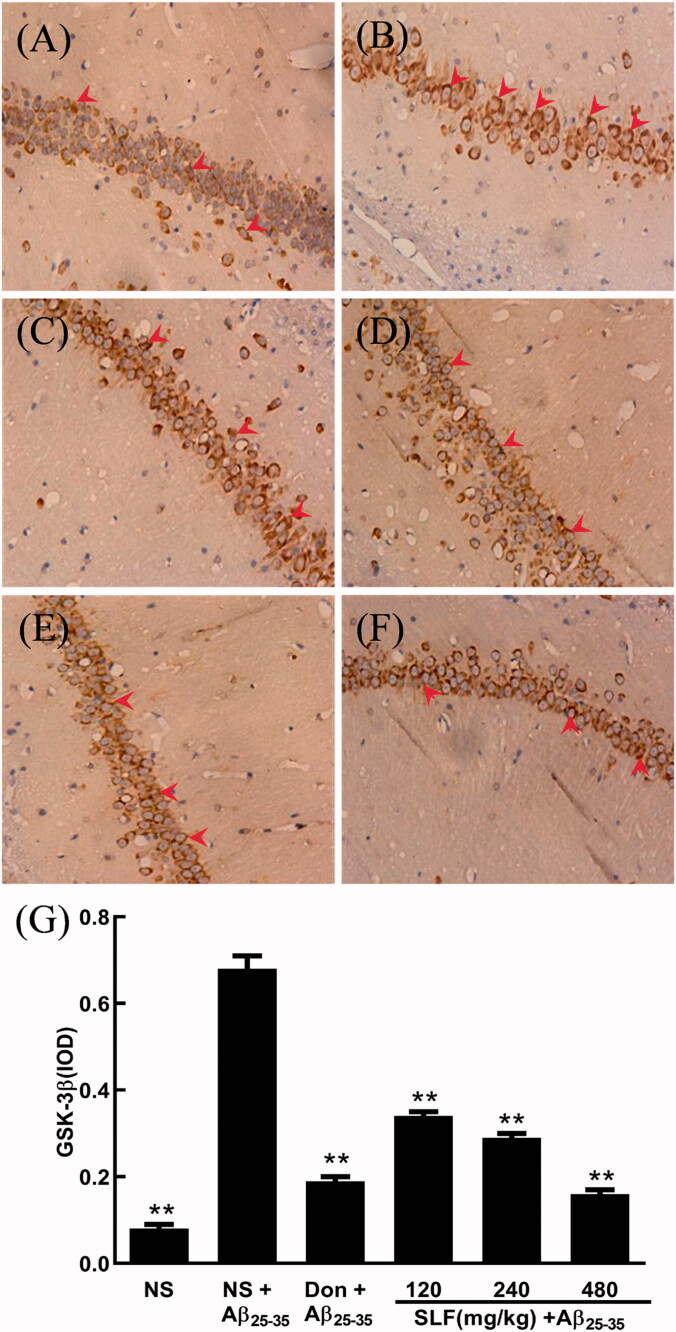
SLF downregulated GSK-3β protein expression in the hippocampal CA1 area of rats with Aβ_25-35_-induced AD by immunohistochemistry (×400). (A) Sham rats treated with NS; (B–F) AD rats treated with NS, donepezil 0.42 mg/kg, SLF 120, 240 and 480 mg/kg by intragastric administration. Ten rats were allocated to each group. The effects of SLF on the integrated optical density of GSK-3β expression in AD rats (G). The results are expressed as mean ± SD (*n* = 10). **p < 0.01, compared to the NS plus Aβ_25-35_ group (normal saline treatment).

The AKT/GSK3β signalling pathway is critical in the cascade of events that culminate in AD as it is involved in the mechanisms of learning and memory (Hu et al. 2013). We used immunohistochemistry and the anti-GSK-3β antibody to determine the protein expression of GSK-3β in the hippocampal CA1 area of experimental rats. As shown in Figure 4(B), a higher level of the positive features of GSK-3β expression was clearly observed in rats with AD, and the IOD of GSK-3β expression was evidently increased compared to that of sham rats (*p * < 0.01; Figure 4(G)). Nonetheless, the number of brown particles significantly decreased after donepezil (Figure 4(C)) or SLF (Figure 4(D–F)) treatment in a dose-dependent manner (*p * < 0.01). In addition, the same trend as the IOD of GSK-3β expression was identified (Figure 4(G)).

### SLF reduced the protein expression of tau in AD rats by immunohistochemistry

**Figure 5.**
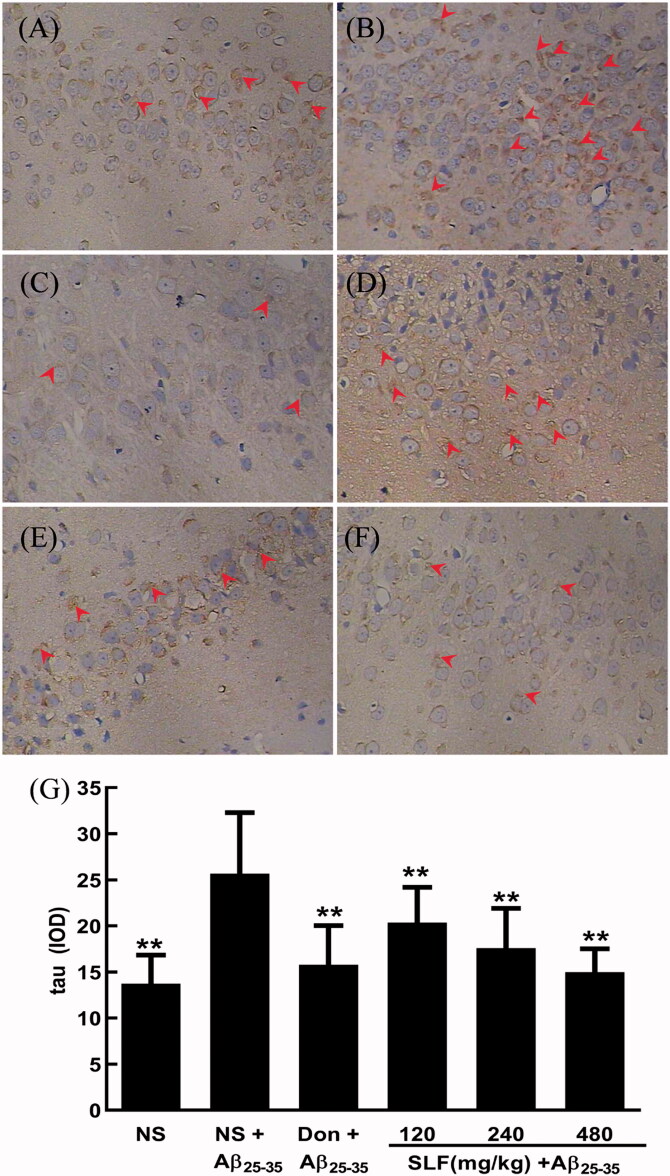
SLF downregulated tau protein expression in the hippocampal CA1 area of rats with Aβ_25-35_-induced AD by immunohistochemistry (×400). (A) Sham rats treated with NS; (B–F) AD rats treated with NS, donepezil 0.42 mg/kg, SLF 120, 240 and 480 mg/kg by intragastric administration. Ten rats were allocated to each group. Effects of SLF on the integrated optical density of tau expression in AD rats (G). The results are expressed as mean ± SD (*n* = 10). **p < 0.01, compared to the NS plus the Aβ_25-35_-group (normal saline treatment).

The high expression of tau protein is an important feature in AD and results in potent neurotoxicity. Therefore, we used the anti-tau antibody to detect the protein expression of tau in the hippocampal CA1 area of experimental rats by immunohistochemistry. As shown in Figure 5(B), a higher level of positive features of tau expression was evident in rats with AD, and the IOD of tau expression was evidently increased relative to that of sham rats (*p* < 0.01; Figure 5(G)). However, the number of brown particles significantly decreased after donepezil (Figure 5(C)) or SLF (Figure 5(D–F)) treatment in a dose-dependent manner (*p* < 0.01), and displayed the same trend as the IOD of GSK-3β expression (Figure 5(G)).

**Figure 6.**
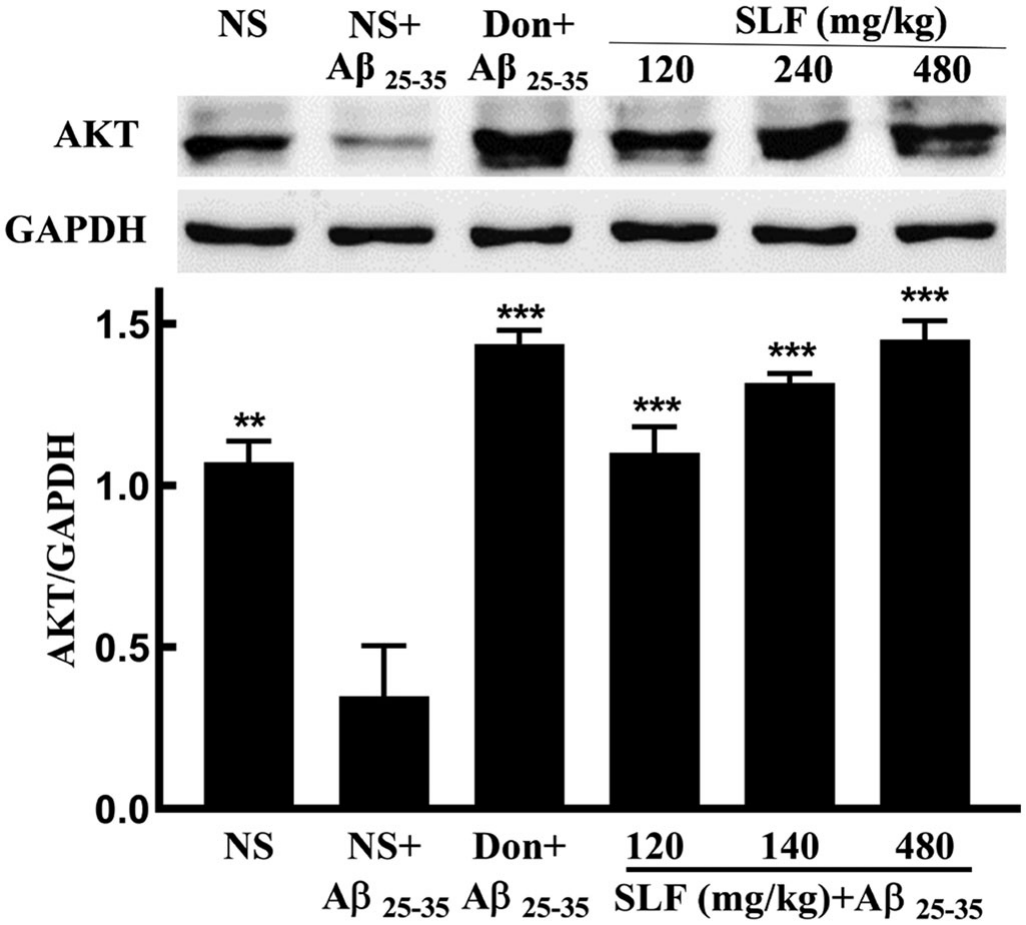
Effects of SLF on AKT protein expression in the hippocampal CA1 area by Western blotting analysis. The results represent at least three independent experiments ran in triplicate and are expressed as mean ± SD. **p < 0.01, ***p < 0.001 vs. NS plus the Aβ_25-35_-treated group.

To confirm the low expression of AKT in AD rats, we performed WB analysis. The result showed that the protein expression level of AKT was significantly downregulated (Figure 6). However, treatment with SLF at concentrations of 120, 240 and 480 mg/kg significantly upregulated the expression level of the AKT protein (*p* < 0.001; Figure 6) in rats treated with Aβ_25-35_.

### SLF decreased the protein expression of GSK-3β in AD rats by WB

**Figure 7.**
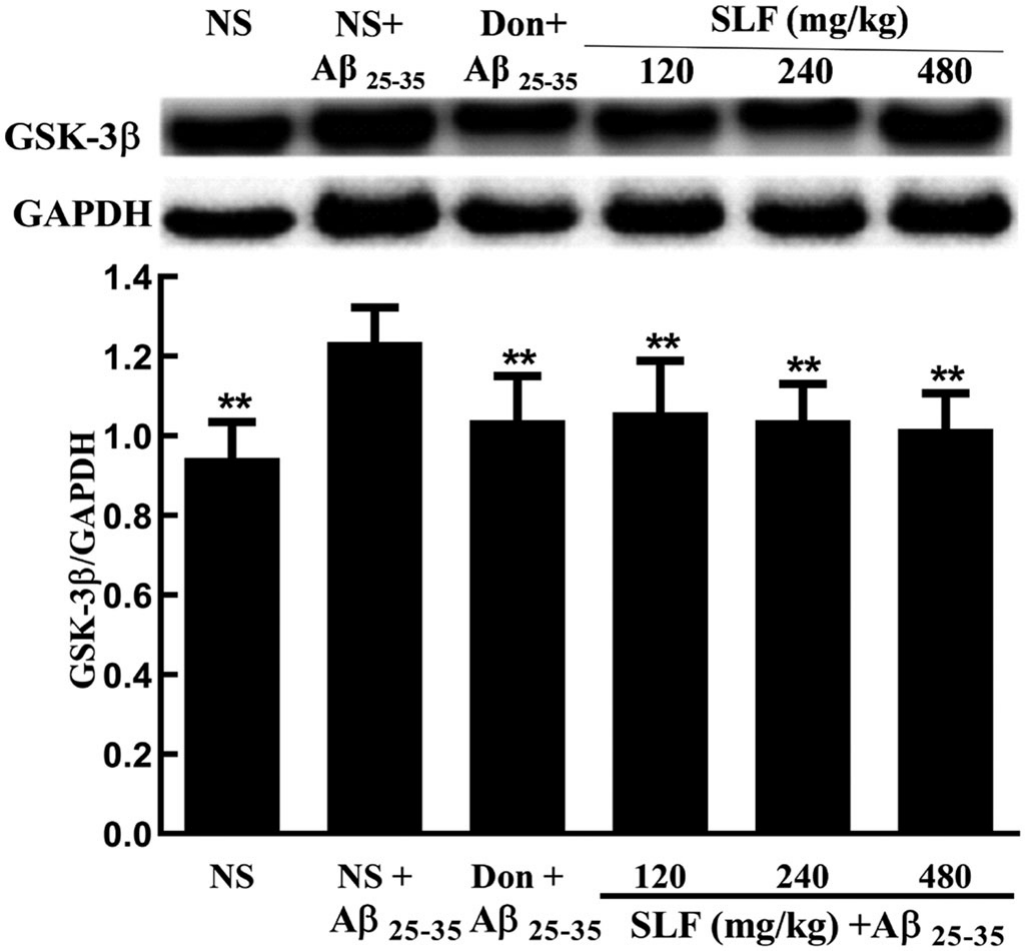
Effects of SLF on GSK-3β protein expression in the hippocampal CA1 area by Western blotting analysis. The results represent at least three independent experiments ran in triplicate and are expressed as mean ± SD. **p < 0.01 vs. NS plus Aβ_25-35_-treated group.

To confirm the high expression of GSK-3β in AD rats, we employed WB to analyse GSK-3β protein expression in AD rats. Based on the results, GSK-3β was significantly upregulated (Figure 7); however, treatment with SLF at concentrations of 120, 240 and 480 mg/kg significantly downregulated the expression level of the GSK-3β protein (*p* < 0.01; Figure 7) in rats treated with Aβ_25-35_.

## Discussion

**Figure 8.**
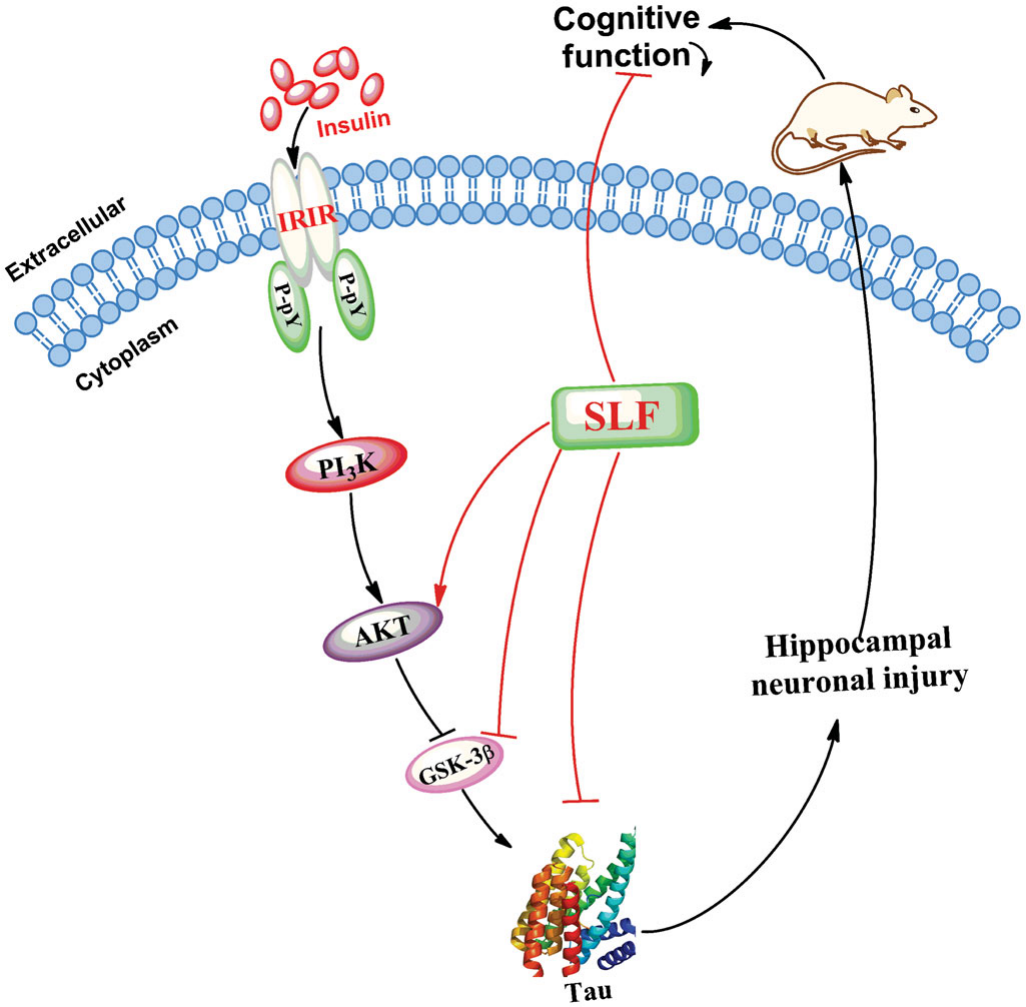
SLF exerts preventive effects in AD by activating the PI3K/AKT/GSK-3β signalling pathway.

AD is clinically characterized by the deterioration of learning, memory and other higher cognitive functions and Aβ plays a crucial role in the pathological process involved in the PI3K/AKT/GSK-3β pathway (Qi et al. 2017; Feng et al. 2018). In the present study, we investigated the effect of SLF on cognitive function *in vivo*. In addition, the protein expression of AKT, GSK-3β and tau in the hippocampus neurons was investigated by immunohistochemistry and WB. Our results revealed that SLF can improve spatial learning and memory, increase the number of rats crossing the platform and the platform quadrant dwell time, enhance the total distance covered on the run platform quadrant in rats with AD, promote AKT protein expression, and decrease the protein expression of GSK-3β in the hippocampus. Therefore, our findings support the proposal that SLF has a potent preventive effect on AD rats via its activation of the PI3K/AKT/GSK-3β signalling pathway (Figure 8). More importantly, SLF may be developed as a natural drug for AD treatment. However, its pharmacological activity and pharmacokinetics in different animal models of AD, as well as its toxic effects, preparation, stability, quality control measures, etc. should be demonstrated before clinical trials for validation.

Phosphatidylinositide 3-kinases (PI3Ks) is a family of enzymes involved in cellular functions such as cell proliferation, growth, differentiation, survival, motility and intracellular trafficking (Bao et al. 2013; Sun et al. 2019). PI3K is also a key component of the insulin signalling pathway and is involved in the PI3K/AKT/GSK-3β pathway, which regulates glucose uptake, learning and memory (Wang et al. 2013). PI3K can be activated by TNF-α through IKK-β to be relative to the inflammation network. Hence, we examined the downstream proteins of the PI3K/AKT/GSK-3β pathway associated with learning and memory and then evaluated and confirmed the protein expression of AKT, GSK-3β and tau by immunohistochemistry and WB. The results indicated that the PI3K/AKT/GSK-3β pathway can be activated by SLF in Aβ_25-35_-induced AD rats.

Survival of the hippocampal neurons is crucial in the pathological process of AD, and the PI3K/AKT/GSK-3β pathway and the associated regulation are involved in this process (Li et al. 2013). Accumulating evidence has, however, revealed that many inflammatory factors, including NF-κB, Forkhead family, P53 and procaspase-3, participate in the pathological process of neuronal apoptosis (Tomek et al. 2012; Wu et al. 2019). The phosphorylation degree and speed of AKT, GSK-3β and tau are important in the execution of this function but is rather difficult to carry out *in vivo*. Therefore, we sought to further investigate the molecular mechanisms associated with the preventive effects of SLF on AD by determining the phosphorylation degree and speed of AKT, GSK-3β and tau within primary cultured hippocampal neuron after the PI3K/AKT/GSK-3β pathway, and the preventive effect of neuronal apoptosis and the associated mechanisms via the inflammatory pathway.

## Conclusions

To the best of our knowledge, the present study is the first to report that SLF can significantly improve cognitive function, such as spatial learning and memory, in rats with AD. We also recognized an obvious upregulated expression of the AKT protein and a decrease in the protein expression of GSK-3β in the hippocampal CA1 area. These results indicate that SLF improves cognitive function and prevents hippocampal neuronal injury in rats with Aβ_25-35_-induced AD via the AKT/GSK-3β signalling pathway. Such findings may provide important insights for the potential discovery and development of SLF as a novel therapeutic medicine; however, this requires further research.
